# Early Left Ventricular Thrombus Formation in a COVID-19 Patient with ST-Elevation Myocardial Infarction

**DOI:** 10.1155/2020/8882463

**Published:** 2020-07-18

**Authors:** Harish Sharma, Sudhakar George

**Affiliations:** ^1^Institute of Cardiovascular Sciences, University of Birmingham, Birmingham, UK; ^2^University Hospitals Birmingham NHS Foundation Trust, Mindelsohn Way, Birmingham B15 2TH, UK

## Abstract

**Background:**

Left ventricular thrombus (LVT) is a complication of acute myocardial infarction (AMI) due to localised haemostasis. LVT is typically seen 3-12 days following AMI and is seldom seen within the first 24 hours. LVT increases the risk of mortality due to systemic thromboembolism. Patients with Coronavirus Disease-19 (COVID-19) are potentially hypercoagulable and this may promote early development of LVT.

**Case:**

A 50-year-old man with no past medical history was admitted with a severe diabetic ketoacidosis following a 4-day history of cough and fever. The patient tested positive for COVID-19 and required intensive care treatment for ventilation and haemofiltration. After returning to ward-based care, the patient developed chest pain and electrocadiographic changes consistent with an acute anterior ST-elevation myocardial infarction. Emergency percutaneous coronary intervention was performed to the left anterior descending artery. However, the patient developed diuretic-resistant pulmonary oedema and a bedside echocardiogram revealed significant LVT despite only 4 hours of chest pain. The thrombus was associated with the anteroseptal wall of the left ventricle which was hypokinetic but not aneurysmal. An intra-aortic balloon pump (IABP) was inserted, but the patient developed ipsilateral lower limb ischaemia due to the formation of thrombus in the femoral artery and irreversible cardiogenic shock from which he ultimately succumbed.

**Conclusion:**

COVID-19-positive patients are potentially hypercoagulable, and MI in this population may precipitate LVT earlier than expected. Consideration should be made for routine early screening of post-MI COVID-19 patients for LVT. If detected, anticoagulation may reduce the risk of cardiovascular mortality in this high-risk group.

## 1. Introduction

Left ventricular thrombus (LVT) complicates up to 15% of all acute myocardial infarction (AMI) cases [1] and 25% of patients with anterior myocardial infarction [2]. A postinfarction triad of hypercoagulability, left ventricular (LV) hypokinesia or aneurysm, and local myocardial inflammation creates an area of relatively static blood which is prone to thrombus formation.

Traditionally, LVT has been detected by transthoracic echocardiography (TTE) with the use of contrast enhancement if the LV endocardium is not clearly visualised. A serial study using TTE found that LVT is typically detected 3-4 days following myocardial infarction (MI), with very few cases detectable within the first 24 hours [3]. In another serial TTE study of 105 patients, no cases of LVT were observed within the first 11.3 ± 8 hours and the median time to the first discovery of LVT following anterior MI was 6 days. Cardiovascular magnetic resonance (CMR) imaging has superseded echocardiography as the most accurate modality for the detection of LVT [4]. Recent evidence has shown that CMR performed 9-12 days post-MI gives the highest detection rate [5].

The incidence of LVT has fallen significantly since widespread early percutaneous coronary intervention (PCI) has been widely adopted for the management of AMI. In clinical practice, LVT is most commonly seen in late-presenting MI patients. In patients who present early and undergo prompt revascularisation, LVT is rarely observed, particularly within the first 24 hours of AMI.

The emergence of the novel zoonotic Severe Acute Respiratory Syndrome Coronavirus-2 (SARS-CoV-2) has resulted in Coronavirus Disease 2019 (COVID-19) becoming a major global pandemic. Although the predominant symptoms are respiratory, cardiovascular complications have also been described. Studies have reported that COVID-19 patients are subject to a significant derangement of haemostasis, resulting in hypercoagulability as seen on whole blood thromboelastography [6].

In this case, we describe a COVID-19 patient who suffered an acute anterior myocardial infarction and had readily detectable, established LVT on transthoracic echocardiography, within 4 hours of presentation.

## 2. History of Presentation

A 50-year old man with no past medical history was admitted to hospital by ambulance after his wife found him unresponsive. On admission, the patient was confused and agitated with a Glasgow Coma Scale (GCS) score of 11.

## 3. Examination

On examination, the patient had mottled legs, dry mucous membranes, and peripheral circulatory collapse. Blood pressure (BP) and peripheral oxygen saturations were unrecordable.

## 4. Investigations

Arterial blood gas measurements revealed a blood glucose beyond the upper limit of recordable value (despite no history of diabetes) and ketones of 3.6 mmol/L with a lactic acidosis.

Notable abnormal blood tests included an acute kidney injury (urea 41.3 mmol/L, creatinine 277 *μ*mol/L, estimated glomerular filtration rate of 21 mL/min), hypernatraemia (Na 172 mmol/L), and hypercholersterolaemia (total cholesterol 9.0 mmol/L). The white cell count was normal (9.3 × 10^9^), but the C-reactive protein level was raised at 53 mg/L and over the following 5 days increased to 374 mg/L.

## 5. Differential Diagnosis

The patient was thought to have a new diagnosis of diabetes mellitus presenting as either a diabetic ketoacidosis or hyperosmolar hyperlycaemic state provoked by COVID-19 infection.

## 6. Course of Disease and Management

The patient was initially treated with intravenous (IV) fluids, antibiotics, and an insulin infusion until the blood glucose reduced to acceptable levels, following which he was treated with subcutaneous insulin. A collateral history revealed that the patient had a 4-day history of cough and pyrexia. One week prior to admission, two members of his household had been unwell with COVID-19 symptoms. The patient tested positive for COVID-19, and despite 3 days of ward-based treatment, his condition continued to deteriorate. Worsening respiratory failure necessitated transfer to the intensive care unit (ICU) for intubation and ventilation. The circulation was supported by vasopressors and continuous veno-venous haemofiltration was commenced.

**Figure 1:**
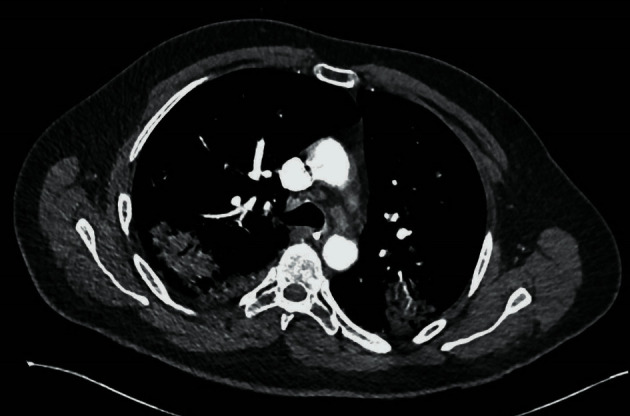
CTPA: bilateral patchy consolidation in keeping with COVID-19 with superadded aspergillosis infection causing cavitating lesions.

Twelve days after admission, the patient was transferred back to the ward. Once again, his respiratory status declined and a computed tomography pulmonary angiogram (CTPA) revealed patchy consolidation and cavitation (Figure 1). Two sputum samples tested positive for aspergillus, and the patient was treated with voriconazole.

**Figure 2:**
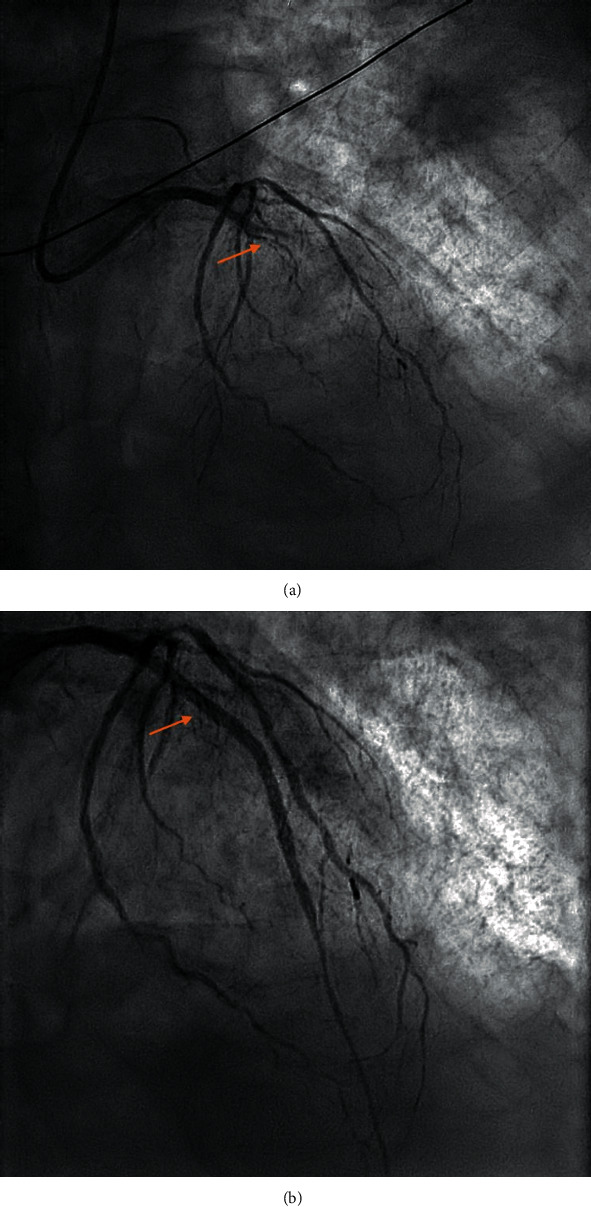
Coronary angiogram in the PA cranial view demonstrating (a) LAD occlusion before intervention and (b) reperfusion following implantation of a drug-eluting stent.

**Figure 3:**
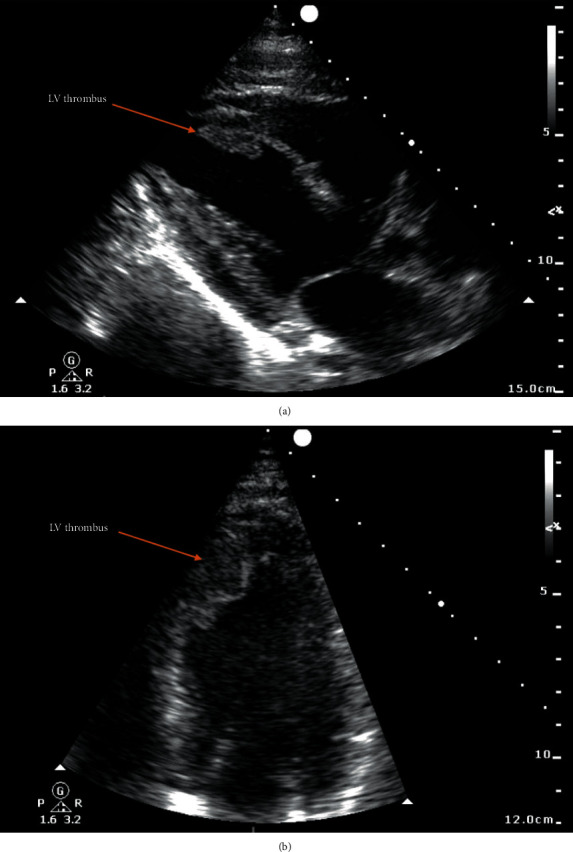
Transthoracic echocardiogram demonstrating thrombus on the mid-to-apical anteroseptal left ventricular wall in the (a) parasternal long axis view and (b) apical 4-chamber view.

One month after admission, the patient suddenly developed acute chest pain. An electrocardiogram (ECG) demonstrated anterior ST-segment elevation. The patient was taken to the cardiac catheterisation suite for an emergency coronary angiogram. This demonstrated acute occlusion of the left anterior descending (LAD) artery (Figure 2(a)) and moderate bystander atheroma in the other coronary vessels. The patient was treated with primary percutaneous coronary intervention (PCI) to the LAD with implantation of a single drug-eluting stent, restoring TIMI 3 flow in the vessel. However, during the procedure, the patient developed acute pulmonary oedema with poor response to IV furosemide. Bedside transthoracic echocardiography revealed moderately impaired left ventricular (LV) function with the greatest function in the basal segments. Surprisingly, LV thrombus was noted attached to the anteroseptal LV wall, which was hypokinetic but not aneurysmal (Figure 3).

The patient was reintubated and readmitted to the intensive care unit. High-sensitive troponin I was measured at >50,000 ng/L one day postinfarction, having been only 2540 ng/L one day earlier, indicating an acute large territory myocardial infarction had occurred. The total plasma cholesterol level was rechecked and was within the normal range. The patient developed cardiogenic shock resistant to inotropes, and despite insertion of an intra-aortic balloon pump (IABP), cardiac output did not improve. Ipsilateral lower limb ischaemia developed as a result of a hypercoagulable state and IABP. Ultrasound of the right femoral artery confirmed the presence of thrombus in the vessel. Vascular surgeons did not feel that the patient would survive emergency vascular surgery, and after discussions with the family, treatment was withdrawn and the patient succumbed to cardiogenic shock.

## 7. Discussion

This case report describes an unusual case whereby established LVT was detected only 4 hours after the onset of acute anterior ST-elevation MI in a patient recovering from severe COVID-19 infection.

Studies have demonstrated that COVID-19 infection is associated with several cardiovascular complications including myocarditis, arrhythmias, and vascular inflammation. Localised inflammation at the plaque level may disrupt stable coronary atheroma and predispose to acute coronary syndromes [7].

Although the patient in this case did not have a history of angina, moderate multivessel coronary atheroma was found on coronary angiography, indicating preexisting asymptomatic coronary artery disease. The presence of COVID-19 infection may have not only promoted plaque instability in the LAD but also the development of early LVT.

Although the prevalence of LVT has fallen in the modern era of early revascularisation, frequency of detection is likely to increase during the COVID-19 pandemic. Firstly, patients with MI symptoms are more likely to avoid early hospital admission due to a fear of contracting COVID-19. Secondly, COVID-19 infection has been associated with markedly hypercoagulable thromboelastography profiles, increasing the risk of early LVT formation.

Detection of LVT in COVID-19 post-MI patients is paramount due to the risk of systemic thromboembolism, further increasing the risk of morbidity and mortality in this high-risk group. International guidelines recommend anticoagulation if LVT is detected [8]. This case highlights that COVID-19 patients presenting with MI are at risk of early LVT formation and should be evaluated with early inpatient echocardiography, with a low threshold for contrast echocardiography or CMR if necessary.

## Additional Points

*Learning Objectives*. Case: a patient with COVID-19 who developed early LV thrombus. (i) Patients with COVID-19 are potentially hypercoagulable and may form LV thrombus far earlier than expected. (ii) Consider early inpatient imaging following acute MI in COVID-19 patients with echocardiography or CMR if necessary.
